# A survey for antibodies against *Fasciola hepatica* in cattle and sheep in Finland indicates a low level of exposure

**DOI:** 10.1186/s13028-023-00688-9

**Published:** 2023-06-22

**Authors:** Heini Gröning, Antti Oksanen, Teresa Skrzypczak, Tiina Autio

**Affiliations:** 1 Animal Health Diagnostic Unit, Laboratory and Research Division, Finnish Food Authority, Neulaniementie 4, Kuopio, FI-70210 Finland; 2 Animal Health Diagnostic Unit, Laboratory and Research Division, Finnish Food Authority, Elektroniikkatie 3, Oulu, FI-90590 Finland; 3 Animal Health Diagnostic Unit, Laboratory and Research Division, Finnish Food Authority, Mustialankatu 3, Helsinki, FI-00790 Finland

**Keywords:** Bovine, Bulk tank milk, ELISA, Liver fluke, Ovine

## Abstract

**Background:**

Fasciolosis is a parasitic infection caused by the liver fluke *Fasciola hepatica* that can have a major economic impact on livestock industry. The prevalence of the disease has recently been increasing in many North European countries. The objective of this study was to determine the prevalence of antibody against *F. hepatica* in Finnish cattle herds and sheep flocks during 2019 by using a commercial enzyme-linked immunosorbent assay (ELISA). Randomly selected bulk tank milk samples were obtained from 660 dairy herds. Blood samples were collected at slaughterhouses from 1944 suckler cows from 309 herds and from 1120 sheep from 95 flocks.

**Results:**

Antibodies against *F. hepatica* were found in 0.45% (95% confidence interval (CI): 0.15–1.33) of the dairy herds and 0.97% (95% CI: 0.33–2.82) of the suckler cow herds. The seropositive herds were located in eastern and central Finland. None of the sampled sheep flocks tested had antibodies against *F. hepatica* (95% CI: 0–3.89). The results of the assays were compared with meat inspection data received from the slaughterhouses. All positive herds also had liver condemnations due to *F. hepatica* based on the meat inspection reports.

**Conclusions:**

Compared to other North European countries, the prevalence of fasciolosis in Finland can be considered low, and according to meat inspection reports, there are no indications of the prevalence increasing in Finland.

## Background

The common liver fluke, also known as the sheep liver fluke (*Fasciola hepatica*), is a trematode parasite that infects a wide range of hosts, including ruminants, horses, rabbits and humans [1]. It causes the parasitic disease fasciolosis and occurs worldwide [1, 2[. The infection is frequently subclinical and can cause production losses through liver condemnations [2] and a reduction in growth, milk yield and fertility [3].

**Fig. 1:**
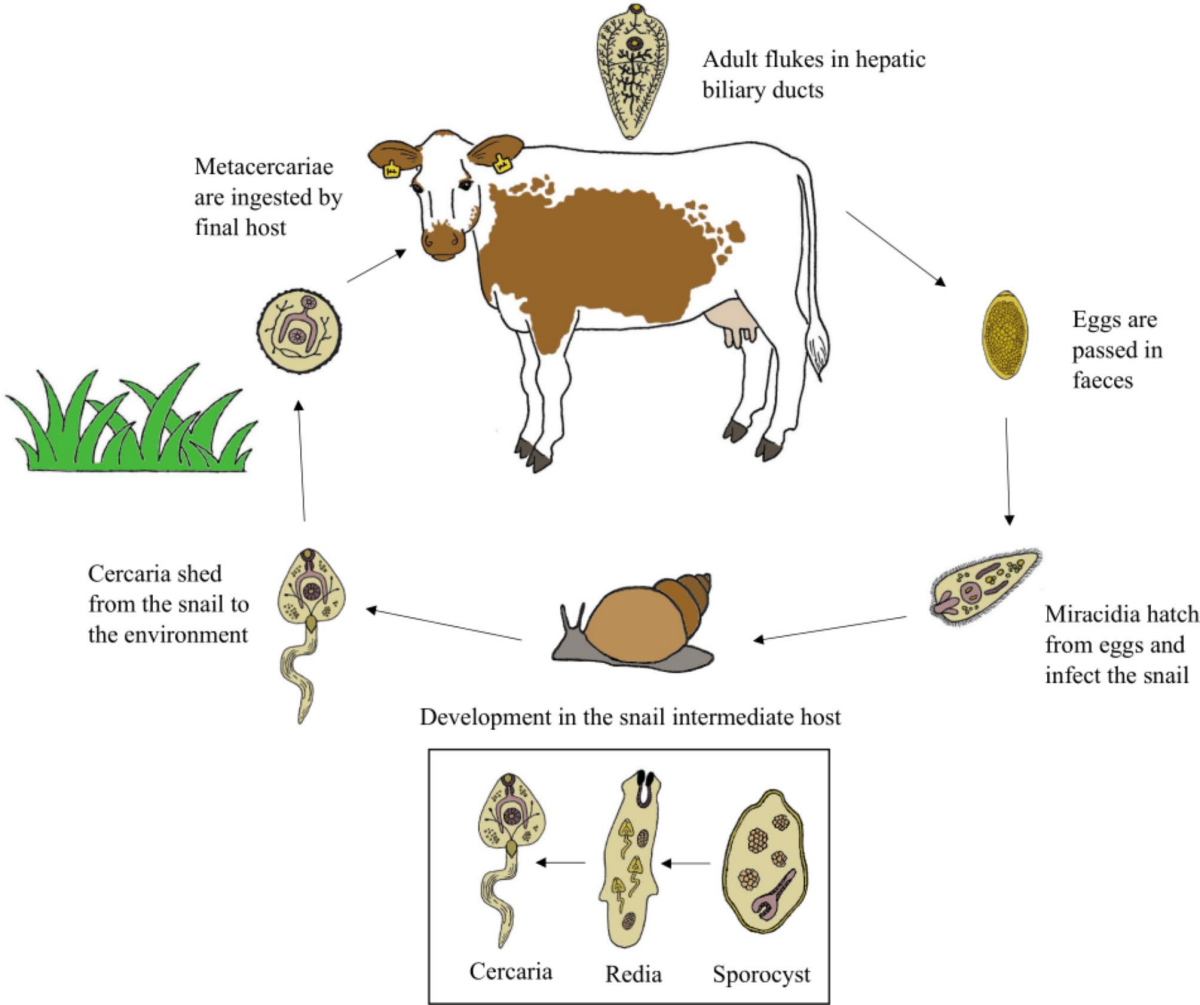
Life cycle of *Fasciola hepatica*

The main intermediate host of *F. hepatica* in Europe is the lymnaeid snail *Galba truncatula*, although over 20 snail species have been reported as alternative intermediate hosts worldwide [4]. The survival of the snail and consequently the life cycle of *F. hepatica* is highly dependent on climatic conditions, such as temperature and moisture [1, 2]. The successful development and reproduction of the parasite in the snail host requires an average daily temperature above 10 °C [5]. After hatching from eggs, miracidia larvae can infect the snail intermediate host. Hundreds of cercariae can be produced by the parasite in the snail and shed into the environment, where they encyst on aquatic plants as metacercariae. Cattle become infected by ingesting infective metacercariae while grazing. In the final host, the larvae excyst in the small intestine, penetrate the intestinal wall, cross the peritoneum to the liver and migrate for 6–8 weeks through the liver parenchyma to biliary ducts, where they mature [1]. Eggs produced by the adult flukes can be detected after 8 weeks of infection, first in the bile and subsequently in the faeces of the final host [4]. If left untreated, the fluke can have a life span of at least nine months, or even much longer in sheep [6]. The life cycle of *F. hepatica* is illustrated in Fig. 1.

The infection pressure in high prevalence areas can be reduced with the systematic use of flukicidal anthelmintics, pasture rotation or a limited grazing period on high-risk pastures [7], fencing off of the snail habitats [8], additional feeding to reduce the proportion of grass in the diet and mowing of pasture [9]. The co-grazing of cattle of different ages could reduce exposure to liver flukes because of the lower egg shedding of older infected animals [10]. In addition, animals that feed on snails, such as ducks or terrestrial snails, could decrease the infection pressure by controlling the number of the intermediate hosts [6, 11, 12].

The diagnosis of fasciolosis is based on liver inspection at an abattoir, faecal egg counts and the detection of specific antibodies in serum or milk by using enzyme-linked immunosorbent assay (ELISA) [3] and copro-antigen ELISA [3, 13]. The detection of specific antibodies against liver flukes in milk or serum samples has been shown to be a sensitive method for diagnosing fasciolosis, especially at the farm-level [1]. Serum antibodies can be detected 2–4 weeks after infection [14–17] and remain high for at least 18 months [16]. After successful treatment, the antibody levels may remain high for months [14, 18]. For this reason, they represent the infection status of the herd rather than the active infection of an individual animal [1]. Compared to faecal egg counts, serology can detect infections 7–8 weeks earlier [15]. Meat inspection is less sensitive than serological methods in detecting infections [13, 19].

All cattle and sheep must be registered in Finland. The Finnish cattle population was ∼860,000, raised on a total of ∼9,800 farms in 2019. The Finnish sheep population was ∼ 145 000, raised on a total of ∼1300 farms in 2019 of which about half were hobby farms. The number of organic farms in 2019 were ∼ 800 and ∼ 185, cattle and sheep farms, respectively. There were 260,000 dairy cows on 6,300 farms. The average herd size was 48 cows among herds in the Finnish Dairy Herd Recording System. The number of dairy farms has decreased during the last 10 years, while the average herd size has increased, and this trend appears to be continuing. There are 60,000 suckler cows on Finnish cattle farms, and 1,600 farms have only suckler cows. The overall density of cattle is rather low, but cattle production is clustered in the central parts of the country. In roughly 70% of Finnish herds cattle graze and grazing season is ∼5 months. Over 95% of dairy herds and 58% of suckler cow herds were included in a voluntary centralized cattle health care register, called Naseva register.

**Fig. 2:**
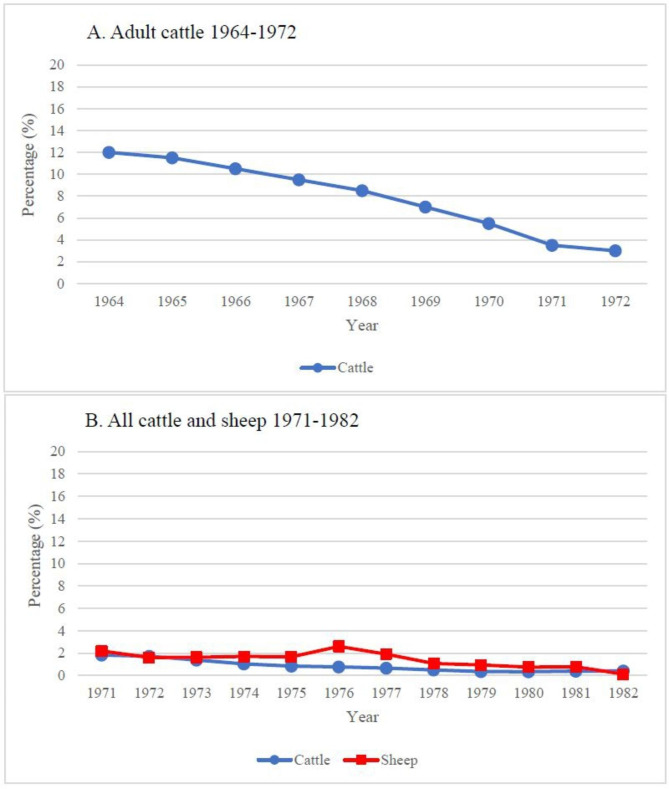
Percentage of meat inspection condemnations due to fasciolosis in Finland: (**A**) Adult cattle 1964–1972 [27] and (**B**) All cattle (blue circles) and sheep (red squares) 1971–1982 [28]

The latest study analysing prevalence of *F. hepatica* in Finland was published in 1974 reporting decreasing prevalence from 12% to 1964 to 3% in 1972 in cattle but showing noticeable differences between regions [20]. National meat inspection statistics showed also a rapid decrease in the prevalence of *F. hepatica* infection in cattle during the 1960–80s (Fig. 2). In 2010s liver condemnation due to parasites has been reported to be even lower (< 0.08%), but the proportion of *F. hepatica* associated liver condemnations has not been specified [21]. In contrary, in the Nordic and Baltic countries, high and increasing herd level prevalence has been reported in the past decades yielding 20–50% prevalence in dairy herds [22–26]. Due to this there was a concern of possible increasing prevalence also in Finland, especially as meat inspection has shown to be less sensitive than serological methods in detecting infections [13, 19].

Fluke related liver condemnation is rare in Finland and in order to get additional support for the assumption of the rarity of *F. hepatica*, we wanted to use another method independent from liver lesions to assess the prevalence. An additional aim was to evaluate the distribution of F. *hepatica* in the country. This knowledge is essential for developing surveillance and assessing the possible need for control measures, such as the implementation of a control programme.

The objective of this study was to determine the prevalence of antibodies against *F. hepatica* in Finnish cattle herds and sheep flocks during 2019 by using a commercial ELISA.

## Methods

### Sample selection and collection

In the monitoring of various bovine diseases in Finland, there is a long tradition of sampling in dairies and slaughterhouses. Herds are sampled according to an annual nationwide plan of monitoring programmes for animal diseases, including the surveillance of brucellosis, infectious bovine rhinotracheitis (IBR) and bovine viral diarrhoea (BVD). Samples taken during December 2018 to December 2019 for surveillance purpose were analysed for the presence of antibodies against *F. hepatica*. Briefly, a total of 660 randomly selected dairy herds, selected from all Finnish cattle herds delivering milk to dairies, were examined using bulk tank milk (BTM) samples taken at dairies. Randomization was done using computer-generated random numbers. Blood samples from randomly selected suckler cow herds (beef cattle, not milked) and sheep flocks were obtained in slaughterhouses. Random selection of suckler cow herds was performed from all Finnish cattle herds having over 24-month-old suckler cows and not having any dairy cows. Randomization was done using computer-generated random numbers. Sampling requests were marked in Bovine Register for each animal of the farm selected for sampling. The slaughterhouse must submit a register query to the Bovine Register before animals are delivered to the slaughterhouse to verify that the animals are registered and are not subject to movement restriction. In the register query also the sampling requests are reviewed. Random selection of all Finnish sheep herds having more than 10 ewes was performed and the list of herds selected were sent to slaughterhouses. Randomization was done using computer-generated random numbers. A total of 1944 blood samples from 309 suckler cow herds and 1120 blood samples from 95 sheep flocks were obtained from slaughterhouses. From suckler cows, a total of 1–40 blood samples per herd were obtained, the average being 6.3 samples per herd. From sheep, the corresponding figures per flock were 1–30 obtained blood samples, with 11.4 samples being the average. The BTM samples were collected during February to June 2019, with the majority collected in February (82%). The suckler cow samples were collected almost evenly during December 2018 to December 2019 and the sheep samples during January to December 2019, with the least being collected during the summer months. The collected samples represented 9.8% and 19.7% of dairy and suckler cow herds, respectively, and 7.2% of sheep flocks in Finland in 2019.

### ***Assay of antibodies against*** **F. hepatica** ***in serum and milk samples***

BTM samples were centrifuged at 1600 x g for 15 min, the fat fraction was removed and the non-fat fraction was stored at -20 °C. Blood samples were centrifuged at 1800 x g for 15 min and the sera were collected and frozen at -20 °C. BTM samples were tested individually and blood samples in pools of 10 samples. All the individual samples within the positive pools were re-tested.

The samples were tested for the presence of antibody to *F. hepatica* by using a commercial ELISA test (IDEXX Fasciolosis verification test, IDEXX, Hoofddorp, the Netherlands) according to the manufacturer’s instructions. Briefly, the optical density (OD) of the sample was corrected by subtracting the OD of the negative control and the results were expressed as the sample-to-positive ratio (S/P%). The sample was considered positive if the S/P% was higher than 30 and as negative if the S/P% was equal to or less than 30. The sensitivity and specificity of the test on milk have been reported as 95.0% and 98.2% relative to sera [29] and on blood 97.7% and 99.5% relative to faecal egg counts [30], respectively.

### Meat inspection data

The slaughterhouses were requested in 2020 to deliver information on liver condemnations due to *F. hepatica* for the previous 3–5 years. We received data from the five largest slaughterhouses in Finland. In all, we received data from 677 cattle and none from sheep from the years 2013–2020. The data received was identification numbers of animals with liver condemnation due to *F. hepatica* and the date of slaughter. To compare with antibody data the identification numbers of cattle farm, and birth cattle farm were obtained from Bovine Register.

### Graphics and statistics

**Fig. 3:**
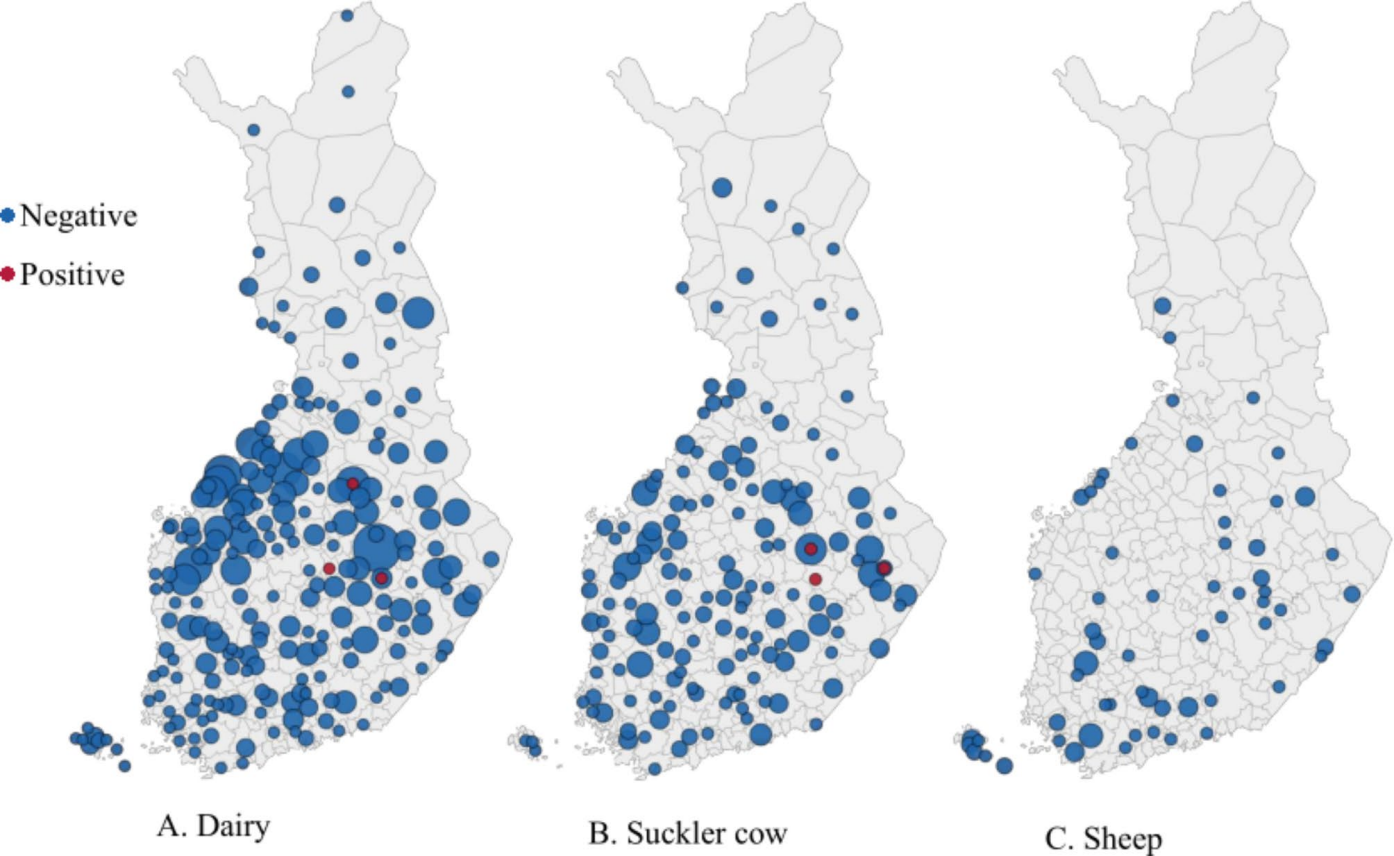
Geographical distribution by municipality of the detected *Fasciola hepatica* antibody negative and positive dairy herds (**A**), suckler cow herds (**B**) and sheep flocks (**C**) in Finland. Negative herds and flocks are marked with blue circles and positive ones with red circles. The size of the circle reflects the number of herds the range being 1–25

Following tools were used for making figures: Fig. 1 GIMP [31], Fig. 2 Microsoft Excel, Fig. 3 Datawrapper [32]. Apparent prevalence of positive herds in the population and the corresponding 95% confidence intervals were estimated using Epitools Epidemiological Calculators [33].

## Results

A total of three dairy herds and three suckler cow herds tested positive for antibodies against *F. hepatica*, yielding an apparent herd-level prevalence of 0.45% and 0.97%, respectively (Table 1). No sheep flocks tested positive for antibodies against *F. hepatica* during the study. The S/P% values for the positive BTM and serum samples were all moderate to high, ranging between 80 and 231.

**Table 1:** Results of survey for antibodies against Fasciola hepatica in Finnish cattle and sheep

Herd category	No. of samples examined	No. of positive samples	No. of herds examined	No. of positive herds	Apparent herd prevalence % (95% CI)
Dairy	660	3	660	3	0.45 (0.15–1.33)
Suckler beef cow	1944	18	309	3	0.97 (0.33–2.82)
Sheep	1120	0	95	0	0 (0–3.89)

The presence of liver flukes in serologically positive herds was confirmed at slaughter during meat inspections. Liver condemnation due to *F. hepatica* were observed in 3–73 animals from all serologically positive suckler cow herds and in 2–20 animals in BTM positive dairy herds (Table 2). There were six herds tested negative in BTM but had cattle with liver condemnation. In two farms these positive animals were not born in the farm and the birth farms were positive for *F. hepatica*. In four cases there was only a single animal with liver condemnation.

**Table 2:** Liver condemnations due to Fasciola hepatica based on meat inspection reports from slaughterhouses (2013–2020) for Finnish cattle herds having antibodies in bulk tank milk (BTM) or serum samples

	Antibodies			Fasciolosis in meat inspection	
	BTM	Serum		Herd status	Seropositive animals
	SP%^a^ of positive samples	No. positive/no. tested		(No. of positive animals 2013–2020)	No. positive/total
**Dairy**					
Herd 1	80			Positive (2)	
Herd 2	152			Positive (20)	
Herd 3	199			Positive (9)	
**Suckler cow**					
Herd 1		7/7		Positive (11)	2/7
Herd 2		10/10		Positive (73)	8/10
Herd 3		1/2		Positive (3)	1/2

^a^SP, sample-to-positive ratio

The cattle herds studied covered all 19 counties of Finland and the sheep flocks were from 16 counties. The positive herds were located in Central Finland, Northern Savonia and North Karelia. Distribution maps of the studied and positive herds and flocks are presented in Fig. 3.

## Discussion

Compared to what has been reported in studies in several other North European countries, the prevalence of *F. hepatica* in Finland remains low. The apparent herd-level prevalence of antibodies against *F. hepatica* of 0.45% in Finnish dairy herds is lower than the 7–25% reported in Sweden [22, 23], 50% in Denmark [26] and 20% in Estonia [24]. In beef cattle herds, the seroprevalence in Finland was 0.97%, which is also low compared to 10% reported in Swedish [34] and 36% in Estonian beef cattle herds [24]. However, the study design, applied ELISA method and definition of a positive herd differ between the studies, making it challenging to compare the results directly.

We did not detect antibodies in samples from sheep flocks in our study and no liver condemnations were reported by the slaughterhouses. However, the number of herds studied was limited and there was no selection based on the age of animals studied. Thus, both young animals and old breeding animals were sampled, which may influence the results. Further studies targeting on sheep grazing on shore pastures should be conducted especially as according to meat inspection reports from 2020 to 2021, the prevalence of liver condemnations in sheep due to changes indicating *F. hepatica* infection in the liver at slaughter was 0.014% and 0.054%, respectively [21].

According to meat inspection reports, there was a steep decrease in the prevalence of *F. hepatica* infection in cattle during the late 1960s (Fig. 2) [20, 28] and according to the latest statistics, the proportion in 2021 was 0.027% [21]. One reason for the decline is suggested to be improvements in livestock husbandry, such as improved grazing using cultivated pastures [20]. Another potential explanation suggested is acid rain during the final decades of the 20th century and its impact on soil, surface water and ecosystems. The peak of acidity in Europe occurred in the 1980s, after which it started to decline as a consequence of international air pollution control measures [27]. Soil and water pH influences the survival and abundance of snails and consequently of liver flukes [2]. The snail intermediate host has controversially been reported to prefer either a slightly acidic or alkaline environment [2, 27], but severe acidity is unsuitable for snail survival [27]. Naturally acidic soil, which is widespread in western Finland [35], together with acid rain might have affected the distribution of *G. truncatula* snails over wide areas, but we are not aware of any published studies on this. Although the acidity caused by acid rain has decreased (SO_2_ emissions in Europe decreased by ~ 80% from 1980 to 2020 [36], the naturally acidic soil in Finland might still be far from ideal for *G. truncatula* survival. The occurrence of liver fluke infections in Finland in both 1964 and 1972 was highest in the eastern parts and lowest in the western parts of the country [20]. The positive herds detected in our study were located in eastern and central Finland.

The time of sampling might affect the level of antibodies to *F. hepatica*, as an infection in summer leads to a peak of antibodies in autumn and winter [17, 37]. Most of the BTM samples in our study were collected during winter and the blood samples around the year. An in-herd antibody prevalence of 12–25% is required to obtain a positive BTM result, meaning that lower rates might remain undetected [26, 38]. The inability to detect herds with a low infection burden in BTM samples should be taken into account when considering the low prevalence of antibodies in dairy herds in Finland. Herds with a low in-herd prevalence could be falsely defined as negative herds, and the *F. hepatica* prevalence in Finnish herds might therefore be higher. However, the low number of liver condemnations due to liver fluke in meat inspection data supports the finding of low prevalence of antibodies in Finland, and it can be assumed that few positive herds remained under the detection limit of the antibody analysis. According to reports from the slaughterhouses, all the studied antibody positive herds also had condemned livers due to liver flukes at meat inspection, indicating that the infections were active at the herd-level. However, according to the meat inspection data, there were also liver condemnations from herds recorded as negative in our study. It seems that these might be explained by animal trade from a positive farm (2 farm out of 6) or they are incorrect records as only single animal per farm had liver condemnation (4 farms out of 6). Another explanation might be lower antibody levels than the detection limit of the BTM sample required in the ELISA test. In our study, the testing of both BTM and blood samples by ELISA did not yield any false positive results, as all the herds with positive ELISA results also had *F. hepatica* findings in meat inspection.

Regardless of the diagnostic method used, the prevalence of *F. hepatica* in Finland was confirmed to be lower than in other North European countries. The climate and housing of the animals in Finland differ considerably from high-prevalence countries, such as Ireland and the United Kingdom, where the wet and mild climate provides a better living environment for the free-living larval stages of *F. hepatica* and the snail intermediate host. Furthermore, the longer pasture season from early spring to late autumn means that animals might be frequently and heavily exposed to *F. hepatica* [37, 39, 40]. In Finland on conventional farming only the dairy cattle and heifers over 8 months old farmed in tie stall are required by regulation to graze or be corralled for 60 days every summer. On organic farms cattle must be grazed daily during grazing period. In ten years, proportion of dairy herds that graze has decreased from 87% (2010) to 72% (2020) and the average grazing season is 4.6 months.

Although it can be assumed that the climate in Finland does not differ very much especially from the northern parts of the other Nordic countries, the prevalence of *F. hepatica* appears to be lower [10, 25]. Therefore, the climate does not fully explain the differences in prevalence. Environmental factors such as shore pastures, herd management and animal trade all additionally have a significant impact on herd exposure to *F. hepatica* [9, 25, 41]. In Finland the acidity of shore pastures in many areas, the short pasture season, rarity of common pastures and the trade of cattle mostly concerning fattening, most likely contribute the low prevalence of the country.

The prevalence of fasciolosis in Finland could possibly increase in the future as a consequence of global climate change, which might expand the habitats of snail intermediate hosts on farms and create more favourable conditions for parasite survival and transmission [2, 3]. Furthermore, the increasingly popular and governmentally subsidized grazing on semi-natural grasslands in Finland might pose a risk of increasing fluke exposure. The soil type and texture, together with slopes in the area, might have an indirect impact on the drainage and vegetation of the pasture and therefore on the suitable wetland habitats for the snail hosts and the prevalence of fasciolosis [9, 39, 42, 43]. An association between higher rainfall and increased exposure to *F. hepatica* has been suggested in several studies [39, 42], although some studies have also confirmed that these only have a minor or inconsistent association with the distribution of *F. hepatica* [34, 44]. According to a study by Munita et al. [44], higher annual rainfall was related to lower antibody levels in BTM, possibly due to the more proactive use of anthelmintics or through the washing effect of the rainfall, resulting in free-living liver fluke stages and snails becoming unavailable to grazing animals. It can be assumed that the lengthening of the thermal summer could cause the distribution of liver flukes to expand in temperate climate zones [45]. A longer summer might also enable an extended grazing season for cattle and thus have an impact on exposure to the parasite and the risk of infection. Milder winter temperatures can also have a positive impact on the survival of snail intermediate hosts during winter months [46]. On the other hand, dry years may cause snail habitats to disappear, leading to reduced numbers of intermediate hosts [44], although the snails can survive in a dry environment by burying into the mud [1].

## Conclusions

In contrast to many other parts of North Europe, where *F. hepatica* has emerged or re-emerged during the last few decades, apparently very low prevalence of *F. hepatica* (< 1%) in cattle and sheep herds was confirmed by serology in Finland. The few positive herds were found in the eastern and central Finland. A steep decrease in the prevalence of *F. hepatica* infection in cattle was reported during the 1960–80s, which was possibly caused by a change in grazing systems, limiting the use of waterfront pastures, and/or acid rainfall, harming the intermediate host. Although the prevalence in Finland is currently low, and according to meat inspection reports there are no indications of it increasing, the development in the near future should be continued to be monitored by meat inspection data to determine whether there will be a similar increase in prevalence to that observed in some European countries. The future development is difficult to predict; the fluke is probably benefiting from climate change and governmentally subsidized grazing on semi-natural grasslands, but the naturally acidic soil in many areas of Finland might restrict the populations of the snail intermediate hosts.

## Data Availability

The datasets used and/or analysed during the current study are available from the corresponding author on reasonable request.

## Declarations

### Prior publication

Data have not been published previously.
